# Impaired Structural Motor Connectome in Amyotrophic Lateral Sclerosis

**DOI:** 10.1371/journal.pone.0024239

**Published:** 2011-09-02

**Authors:** Esther Verstraete, Jan H. Veldink, Rene C. W. Mandl, Leonard H. van den Berg, Martijn P. van den Heuvel

**Affiliations:** 1 Department of Neurology, Rudolf Magnus Institute of Neuroscience, University Medical Center Utrecht, Utrecht, The Netherlands; 2 Rudolf Magnus Institute of Neuroscience, University Medical Center Utrecht, Utrecht, The Netherlands; Beijing Normal University, China

## Abstract

Amyotrophic lateral sclerosis (ALS) is a severe neurodegenerative disease selectively affecting upper and lower motor neurons. Patients with ALS suffer from progressive paralysis and eventually die on average after three years. The underlying neurobiology of upper motor neuron degeneration and its effects on the complex network of the brain are, however, largely unknown. Here, we examined the effects of ALS on the structural brain network topology in 35 patients with ALS and 19 healthy controls. Using diffusion tensor imaging (DTI), the brain network was reconstructed for each individual participant. The connectivity of this reconstructed brain network was compared between patients and controls using complexity theory without - *a priori* selected - regions of interest. Patients with ALS showed an impaired sub-network of regions with reduced white matter connectivity (p = 0.0108, permutation testing). This impaired sub-network was strongly centered around primary motor regions (bilateral precentral gyrus and right paracentral lobule), including secondary motor regions (bilateral caudal middle frontal gyrus and pallidum) as well as high-order hub regions (right posterior cingulate and precuneus). In addition, we found a significant reduction in overall efficiency (p = 0.0095) and clustering (p = 0.0415). From our findings, we conclude that upper motor neuron degeneration in ALS affects both primary motor connections as well as secondary motor connections, together composing an impaired sub-network. The degenerative process in ALS was found to be widespread, but interlinked and targeted to the motor connectome.

## Introduction

Amyotrophic lateral sclerosis (ALS) is a fatal neurodegenerative disease, characterized by selective loss of lower motor neurons in the spinal cord and upper motor neurons in the brain. Patients suffer from progressive paralysis and eventually die from respiratory failure. Besides motor symptoms, a subset of patients develop cognitive disturbances or even frontotemporal dementia (FTD), indicating ALS can involve extra-motor brain regions. The peak incidence of this devastating disease lies between 50 and 75 years of age and the average time of survival is about 3 years after onset of symptoms [1].

The effects of upper motor neuron degeneration on the brain are largely unknown. Enhancing the insight in these degenerative effects of ALS is essential towards better treatment. Conventional magnetic resonance imaging (MRI) of the brain does not show characteristic changes indicating upper motor neuron loss in ALS [2]. Computational MRI analysis techniques have shown to be more promising in demonstrating the degenerative effects of ALS [3], [4], [5]. Previous DTI studies have mainly been focused on the corticospinal tract, the white matter ‘highway’ connecting the upper and lower motor neurons, showing reduced white matter integrity in ALS [6], [7], [8], [9], [10]. In addition, recent studies have consistently demonstrated involvement of the corpus callosum, being the primary intracerebral motor connection [4], [5]. However, the involvement of other intracerebral structural connections has only been partly explored. A small number of DTI studies have included analysis of fractional anisotropy (FA) data in a voxelwise manner showing widespread degenerative effects [11], [12]. It is unknown, however, whether these widespread effects occur independently or linked to each other.

Brain regions are interconnected as a network, thereby strongly influencing each other [13], [14]. A mathematical framework to examine the topology of complex network systems has been adopted to study the organization of these connections [15], [16], [17]. Modern neuroimaging techniques, e.g. DTI, permit reconstruction of white matter tracts of the human brain [18]. Studies using complexity theory – like graph theory - demonstrated that the organization of the brain network or connectome plays a crucial part in healthy brain functioning [19], [20], [21].

As the brain is a complex system of interacting regions, local degeneration of upper motor neurons in ALS may have a widespread effect on the brain network. Here, by combining DTI and graph theory, we examined the integrity of the structural brain network in patients with ALS to provide further insight in whether the degenerative effects of ALS occur independently or as a connected system.

## Methods

### Ethics statement

The medical ethics committee for research in humans of the University Medical Center Utrecht, the Netherlands has approved this research. Informed written consent was obtained from all participants. All clinical investigation has been conducted according to the principles expressed in the Declaration of Helsinki.

### Participants

Thirty-five patients with ALS (mean age 50.8; SD 13.0 years; 28 males and 7 females) and 19 age-matched healthy control subjects participated in this study (mean age 53.1; SD 10.5 years; 14 males and 5 females). Patients diagnosed with ALS according to the El Escorial criteria were recruited from the ALS outpatient clinic of the University Medical Center, Utrecht. The clinical characteristics are listed in Table 1. No patients fulfilled the clinical criteria of FTD [22]. Subjects with a history of brain injury, epilepsy, psychiatric illness and other neurodegenerative diseases were excluded, resulting in the group of participants described. Clinical status of the patients was evaluated using the ALS Functional Rating Scale-Revised (ALSFRS-R). The ALSFRS-R is a validated rating instrument for monitoring the progression of disability in patients with ALS [23]. Disease progression rate was calculated, defined as the average decline in ALSFRS-R-score since disease onset ((48 - ALSFRS-R-score)/disease duration in months).

**Table 1:** Demographic and clinical characteristics of all study participants.

	Healthy controls (n = 19)	ALS patients (n = 35)	
	Mean±SD (range)	Mean±SD (range)	
Age (years)	53.1±10.5 (33-67)	50.8±13.0 (26-78) a	
Sex (male/female)	14/5	28/7 b	
Site of onset (n)		Bulbar	5 (14%)
		Cervical	18 (51%)
		Lumbosacral	12 (35%)
Side of onset (n)		Left	19 (54%)
		Right	9 (26%)
		Symmetrical	7 (20%)
Disease duration (months)		17.7±18.0 (3-59)	
ALSFRS-R		40.1±4.3 (30-47)	
Progression rate		0.6±0.5 (0.1-2.0)	

SD = standard deviation. ALSFRS-R = revised ALS functional rating scale.

a comparison of age between the groups, did not result show any significant differences (t-test statistics, p = 0.49).

b the proportion males and females is not significantly different in patients versus controls (Fisher’s exact test, p = 0.42).

### MRI Acquisition

All participants underwent a 35-minute scanning session, in which Diffusion Tensor Imaging (DTI) - for reconstructing the white matter tracts of the brain network - and T1 images - for anatomical reference - were acquired and manually checked. MRI scans were made on a 3 Tesla Philips Achieva Clinical scanner at the University Medical Center Utrecht using a sixteen channel SENSE receiver head-coil. Within each scanning session, first, 2 DTI sets, each consisting of 30 weighted diffusion scans and 5 unweighted B = 0 scans, were acquired (DTI-MR using parallel imaging SENSE p-reduction 3; high angular gradient set of 30 different weighted directions [4], [14], [24], [25], TR/TE = 7035/68 ms, 2×2×2 mm, 75 slices, b = 1000 m/s, second set with reversed k-space read-out). Directly after the acquisition of the DTI scans, an anatomical T1-weighted image was acquired (3D FFE using parallel imaging; TR/TE 10/4.6 ms; FOV 240×240 mm, 200 slices, 0.75 mm isotropic voxelsize).

### Image preprocessing

#### DTI preprocessing and fiber tracking

The DTI preprocessing and reconstruction of the white matter pathways included the following steps. First, the 5 B = 0 images of each of the two DTI sets were averaged, improving the signal to noise ratio of the B = 0 images. Next, susceptibility distortions were corrected by computing a field distortion map using the two average unweighted B = 0 images, based on the fact that they were acquired with an opposite k-space read-out direction [26]. The resulting field map was then applied to the two B = 0 images and the two sets of 30 weighted images [26], resulting in a single set of 30 weighted directions which were realigned with a corrected B = 0 image [27]. Third, eddy-current distortions, often observed in acquisition of single-shot EPI images, were corrected [27]. Fourth, for each voxel, the diffusion profile was fitted a tensor using a robust tensor fit method based on M-estimators [28]. The principal eigenvector of the eigenvalue decomposition of the fitted tensor was computed, marking the preferred diffusion direction in each voxel. For each voxel the FA was computed [29], [30], with high FA values indicating a preferred, diffusion direction of the water molecules. Next, in the final (fifth) step, for each individual DTI dataset, white matter tracts of the brain were reconstructed, often referred to as *fibers* or *tracts,* using the *Fiber Assignment by Continuous Tracking* (FACT) [31], [32], [33]. Tracking parameters were set as follows: from each white voxel in the brain mask, a single seed was started, following the main diffusion direction of each voxel, traveling from voxel to voxel, reconstructing the white matter fiber step-by-step. Fiber tracking was stopped when the fiber trace reached a voxel with a FA value lower than 0.1, when the trajectory of the traced fiber exceeded the brain mask or when the streamline made a turn of more than 45 degrees. Only streamlines with a length that exceeded 30 mm were considered for further analysis. Furthermore, to indicate the integrity of a reconstructed fiber tract, each point of the fiber tract was colored with the FA values of the voxels along the 3D path of the fiber [14], [34], [35], [36].

#### T1 preprocessing and brain region parcellation

Cortical and sub-cortical brain regions were selected by parcellating the cerebrum into distinct, anatomically separated brain regions on the basis of the T1-weighted image, using the well-validated Freesurfer suite (V4.5, http://surfer.nmr.mgh.harvard.edu/). In short, this included the automatic segmentation of grey and white matter tissue, followed by parcellation of the segmented grey matter mask into distinct brain regions, based on a normalized template, parcellating the brain into a number of brain regions, including left and right caudate nucleus, globus pallidum, nucleus accumbens, thalamus, amygdala, hippocampus and 70 cortical brain regions. In total, 82 distinct brain regions were parcellated [37], [38].

### Construction of structural brain networks

**Figure 1:**
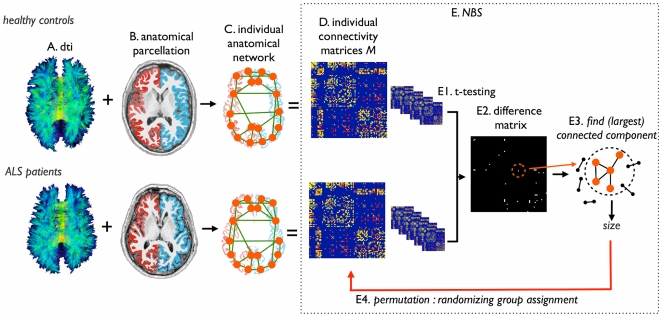
Overview of the network selection procedure and the Network Based Statistics (NBS). (a) Using the DTI data, white matter tracts of the brain were reconstructed. (b) Cortical and sub-cortical brain regions were selected by automatic parcellation of the cerebrum. (c) An individual brain network was defined, consisting of nodes (i.e. the parcellated brain regions) and connections between nodes *i* and *j* that were connected by a white matter pathway. (d) Repeating this for each region *i* and *j* in the collection of parcellated brain regions, resulted in a (weighted) connectivity matrix *M*. Connections were weighted by their FA value, as determined from the DTI measurement. Next, using Network Based Statistics (NBS), the connectivity matrices of ALS patients and controls were compared. (e1) First, each connection between region *i* and *j* was tested between patients and controls using t-statistics. (e2) This resulted in a binary difference matrix, with 1s for those connections that showed a (absolute) t-value between controls and patients higher than a set T-threshold *T*, and 0 otherwise. Third, the sizes of the (largest) connected components in the difference matrix was computed, revealing sub-networks of regions showing affected connectivity in patients. Fourth, permutation testing was used to define a distribution of (largest) component size that could occur under the null-hypothesis (i.e. no difference between patient and controls). 5000 permutations, permuting group assignment, were computed. Finally, the original observed component size (i.e. difference between patients and controls) was given a p-value based on the computed null-distribution, by defining the percentage of the null-distribution that exceeded the size of the observed impaired network in patients.

Using the collection of all reconstructed DTI fiber tracts and the collection of parcellated brain regions, for each individual dataset a structural brain network was constructed, using a validated network framework [13], [19], [39]. This procedure included the following steps, illustrated one-by-one in Figure 1. A graph *G = (V,E)* is a mathematical description of a network, consisting of a collection of nodes *V* and a collection of connections *E*, that interconnect the nodes of the network. Within this framework, the brain network can be expressed as a collection of nodes, reflecting the different anatomical regions and a collection of connections between the nodes, representing the white matter tracts interconnecting cortical and sub-cortical regions. Two nodes *i* and *j* of the network (i.e. brain region *i* and region *j*) were defined as being connected when there was a reconstructed white matter tract in the collection of DTI fibers that interconnected region *i* and region *j*. This selection covered the following specific steps. First, for each individual dataset, the brain regions (i.e. nodes of the *G*) were defined as the individually segmented brain regions. Next, the existence of connections between the nodes (i.e. anatomical brain regions) was computed: for each pair of regions in the individual specific parcellation map it was determined whether the two regions were connected by a white matter pathway [14], [36]. When the fiber selection procedure did not reveal any fibers between region *i* and *j*, no connection was included in *G*. This procedure was repeated for all regions *i* and *j* in the parcellation map. Furthermore, for each connection in *G*, the strength *S* of the connection was taken as its average FA (believed to reflect the level of microstructural organization of the tract). Repeating this procedure for all combinations of regions *i* and *j* in the network resulted in a sparsely weighted connectivity matrix *M*, with weighted connections between those brain regions that are structurally connected and zeros otherwise. Next, to correct for possible individual differences in overall connectivity strength, the connectivity matrix *M* was scaled to the maximum FA value within *M* [25]. As a result, each individual matrix *M* expressed the connectivity structure of the brain network, with the cells of *M* representing the connections and their values representing the connectivity efficacy between brain regions. Please note that, related to the noise of the DTI signal, it is known that streamline tractography may result in the construction of some incorrect streamlines, possibly leading to the inclusion of false positives. However, as a falsely constructed streamline is likely to only occur in a single subject or in a small subgroup across the total group of subjects - otherwise it is more likely to represent a true (i.e. true positive) existing white matter pathway - false positives were controlled for by taking into consideration only those connections that were present in 2/3 of both the patients and controls. Hereby minimizing the effect of incorrect streamlines on the analysis.

### Overall topology of the brain network

Overall topology of the structural brain networks - in both patients and healthy controls - were examined by computing the overall connectivity strength *S* (sum of the connectivity matrix), the average shortest path length *L* (as a measure of global connectivity), the average clustering coefficient *C* (as a measure of local connectivity in the network). Typically *C* and *L* values are normalized to the clustering-coefficient and path length of a collection of random graphs to examine how the graph metrics *C* and *L* relate to the properties of a randomly organized network. To this end, *C* and *L* were compared to the metrics of 100 random graphs with equal degree and degree distribution as the examined brain networks [40]. Furthermore, providing insight in the distribution of connectivity, the average connectivity distribution - i.e. a histogram of how many times a certain level of node-specific strength *S* occurs in the network - of the brain networks of patients and controls was computed. Possible differences in *S*, *C*, *L*, normalized *C* and/or normalized *L* between the two groups were examined using permutation testing (5000 permutations) [25].

### Statistical testing for impaired networks

Next, using the individual brain networks (i.e. matrices *M*) of patients and controls, it was examined whether patients with ALS showed impaired structural connections (i.e. reduced connectivity strength of specific node-to-node connections), compared to the brain networks of the healthy controls. Network Based Statistic (NBS), as proposed by Zalesky et al [41], was used to identify impaired sub-network(s) in patients in comparison to healthy controls. The rationale behind NBS expands the notion of Statistical Parameter Mapping, marking that impaired connections that form a network (i.e. together make up a connected component in the network) have a higher probability of indicating true abnormality, than single connections (i.e. not forming a connected component), hence providing control for the high number of tests performed (i.e. good control for type I error in the problem of multiple comparison). A detailed description of the NBS methodology is given by Zalesky et al. [41]. The performed NBS analysis consisted of the following steps. First, for each connection in the brain network the mean difference in connectivity strength between the group of patients and the group of controls was tested using a two-sample t-test (leaving out zeros). Within the NBS framework, differences in connectivity strength between the group of patients and the group of controls with a t-value larger than a set threshold *T* were marked by 1 in a difference matrix *D,* and 0 otherwise. Within the NBS procedure, the choice of T-threshold is rather arbitrary [41]. However, in this context it is worth mentioning that type I error is always ensured with NBS, irrespectively of the choice of T-threshold as illustrated by simulations performed in the original NBS paper of Zalesky and colleagues [41]. However, type II error (false negative rate) may be impacted by the choice of T-threshold. Therefore, two analyses were performed: (1) using a two-sided T-threshold reflecting a p-value of 0.0075 [41]; (2) using a more exploratory one-sided threshold matching a p-value of 1/N, with *N* the number of nodes in the network. From the resulting matrix *D* the size of the largest interconnected component was computed, marking the size of the cluster of affected connections in the group of patients related to the group of controls. Thirdly, permutation analysis was used to create a distribution (i.e. null-distribution) of component size that can occur under the null-hypothesis. For each of the permutations, (1) subjects (patients and controls) were randomly assigned to two random groups (of similar size as the original patient and control groups); (2) the difference matrix *D* between these two groups was computed; (3) matrix *D* was thresholded; (4) the size of the largest component (i.e. the number of nodes and connections involved) was computed. In the present study, 5000 permutations were performed to create a null-distribution. Fourthly, given the obtained null-distribution, a corrected p-value of the components observed in the original difference matrix *D* between the patients and controls was computed as the percentage of the null-distribution that had a higher value than the observed component size [41].

### Examining the topology of impaired sub-network(s)

The observed impaired structural network(s), expressing reduced connectivity strength in patients compared to controls, was taken for further analyses on topology [25], [40], [41]. For all individual subjects (both patients and controls) a sub-network was formed out of the connections of the reported NBS network(s), by extracting the connectivity values out of the individual connectivity matrices *M*. Next, for each of the individual sub-network(s) the level of connectivity strength *S* was computed, expressing how strong the nodes of the impaired network(s) were interconnected, together with the level of efficiency *E*, expressing the level of efficiency of how each region was connected to other regions in the network. In addition, to *S* and *E*, the level of local clustering (expressed by the clustering-coefficient *C*) was computed, providing information on possible changes in the level of local efficiency or local cliqueness of the affected sub-network [40]. Furthermore, to examine the role of each of the nodes in the impaired sub-network(s), the node-specific connectivity strength *S_i_* and the node-specific efficiency *Eff_i_* were computed. The connection strength *S_i_* of each node *i* expresses how strong a node is connected to the other nodes of the network and was computed by summing up the weights of all its connections. The level of efficiency *Eff_i_* of node *i* was defined as the sum of the inverse distances between node *i* and all other nodes *j* in the network, indicating how efficient information from node *i* can be shared with the other nodes of the network [40].

### Linking graph organizational measures to clinical scores

A possible association between the topology of the impaired network(s) - revealed by the NBS procedure - and clinical scores was explored. ALSFRS-R and disease progression rate was examined against *S_i_* and *Eff_i_* metrics of the impaired network(s) using linear regression.

## Results

### Overall network topology

Table 2 summarizes the global graph metrics of the brain networks of ALS patients and healthy controls, supporting the results of recent studies, indicating a high level of local clustering and a short overall path length [25], [42]. The average connectivity distribution of patients and controls is given in Supplemental Figure S1. As expected, no differences were found in any of the overall graph metrics in ALS (permutation testing, 5000 permutations), suggesting an intact organization of the global brain network in patients.

**Table 2:** Global graph metrics.

	FA weighted brain network	
	Healthy controls	ALS patients
Graph metrics	Mean±SD	Mean±SD
Connectivity strength *S* (sum of *M*)	840±75.8	843±72.8
Shortest path length *L*	3.04±0.18	3.04±0.14
Clustering coefficient *C*	0.37±0.02	0.36±0.02
Normalized characteristic path length	1.11±0.014	1.08±0.017
Normalized clustering-coefficient	2.2±0.16	2.1±0.17

Table summarizes the global values of connectivity strength *S,* shortest path length *L,* normalized path length, and normalized clustering coefficient *C* (normalized to 100 random graphs).

FA = fractional anisotropy. SD = standard deviation.

### Impaired sub-network

**Figure 2:**
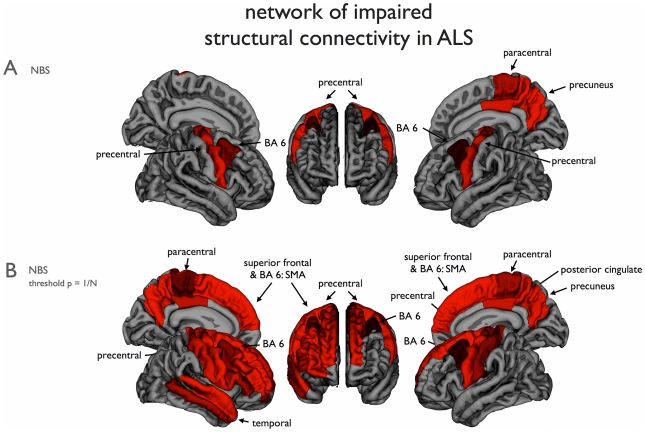
Cortical brain regions with impaired structural connectivity in ALS. (a) The NBS procedure revealed a sub-network of brain regions showing significantly reduced structural connectivity in ALS patients, compared to the healthy controls. Figure shows the set of involved parcellated cortical regions (p = 0.0075, see materials and methods). (b) Using an NBS threshold of p = 1/N (*N* being the number of nodes of the network), a similar but more extended network was revealed. This model-free approach revealed a sub-network consistent with known motor regions, including precentral and paracentral gyri (primary motor), caudal middle frontal and superior frontal gyri (supplemental motor areas, BA6). The subcortical structures found with the NBS procedure were not included in this figure. Right = Right; Left = Left.

**Figure 3:**
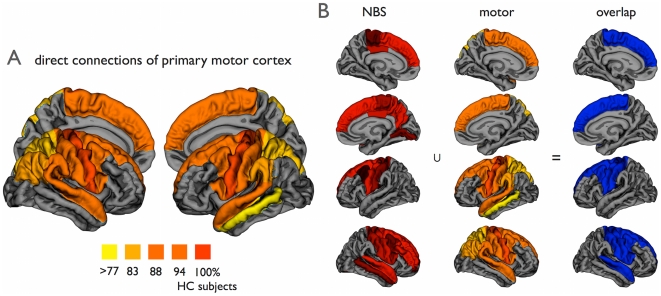
Overlap between motor network and impaired NBS network. (a) Direct cortical connections of the primary motor network. The direct connections of the left and right precentral gyri in the group of healthy controls are shown. Figure illustrates (per region) the percentage of healthy control subjects that showed a direct structural white matter connection to the left or right precentral gyrus. The primary motor network was selected as those regions that were connected to the primary motor regions in the majority of healthy controls (>75%). (b) Figure shows the overlap (right column) between the exploratory NBS network (left column, NBSb network, Figure 2b) and the regions of the healthy motor network (middle column, showing regions of Figure 3a). The impaired NBS network was found to strongly overlap the motor network (p<0.0001, Fisher’s Exact test). Right = Right; Left = Left.

The NBS revealed a single impaired sub-network, consisting of 9 nodes and 10 (bidirectional) connections, of reduced connectivity in patients with ALS (NBSa; p = 0.0108, permutation testing). This impaired network overlapped with the left and right precentral gyrus, left pallidum, left hippocampus, left and right caudal middle frontal gyrus, right paracentral gyrus, right posterior cingulate and right precuneus (Figure 2a and 3). Interestingly, although a whole brain analysis was performed without any a priori selection of motor regions, the regions of this impaired network strongly overlap with regions that are known to play a key role in motor movement and control. To further examine this overlap an additional analysis was performed (see below).

Furthermore, using a NBS-threshold matching a p-value of 1/N, a more extended network was found, including frontal and temporal regions as shown in Figure 2b (NBSb; p = 0.0084, permutation testing). Similar to the more conservative selected network, the sub-network overlapped with the primary motor regions (bilateral precentral gyrus and paracentral lobule), supplemental motor regions (bilateral caudal middle frontal gyrus, superior frontal gyrus and pallidum), but also with a number of other frontal cortical regions (right rostral middle frontal gyrus and right pars triangularis), temporal cortical regions (right middle and superior temporal gyrus), parietal cortical regions (bilateral postcentral gyrus, posterior cingulate and precuneus) and the bilateral hippocampus and right amygdala. The involved cortical regions are shown in Figure 2b. [A post-hoc analysis including age as a covariate in the NBS analysis revealed no fundamental changes in the significance and/or topology of the observed NBS network (Figure 2)].

### Overlap with the motor network

To further examine this consistency between the impaired network and the motor network, we determined the overlap between the regions that form the motor network and the observed impaired sub-network in patients with ALS. The overlap was statistically assessed using Fisher’s exact test. First, the healthy motor network was determined by selecting the (direct) connections of the left and right precentral gyrus - as primary motor regions - with other brain regions in the group of healthy controls. The connectivity matrix *M* of the group of healthy controls was used to extract the direct connections of the left and right precentral gyrus. Figure 3a illustrates the regions that together form the (direct) motor network, i.e. those regions that show a direct connection with the left and right primary motor regions in the majority of the group of healthy subjects. Comparing this motor network to the found impaired sub-network in ALS patients, the NBSa network (Figure 2a) showed large overlap with the motor network. The regions found with the NBSa procedure were 89% motor regions and non-NBSa regions were 66% non-motor regions (Fisher’s exact test: p = 0.0025). Furthermore, the overlap with the extended NBSb network (Figure 2b) was found to be significant as well (p<0.0001); 76% of the NBSb regions were motor regions and 75% of the non-NBSb regions were non-motor (the overlap is illustrated in Figure 3b). Exact numbers of overlapping regions are given in Table 3.

**Table 3:** Overlap between the impaired sub-network in ALS and the motor network.

(Figure 3a)	NBSa	non NBSa
motor	8	25
non-motor	1	48
	*p = 0.0025*	

(Figure 3b)	NBSb	non NBSb
motor	19	14
non-motor	6	43
	*p<0.0001)*	

The overlap of included regions is depicted for both NBS thresholds (see methods). The resulting p-value of the Fisher’s exact test is included.

NBS = network based statistics.

### Topology of the impaired NBS network

Examining the topology of this impaired network (NBSa, Figure 2a) revealed a significantly reduced level of network efficiency *E* (p = 0.0095, t-test, df = 52), as well as a reduced level of overall clustering *C* (p = 0.0415, t-test, df = 52). Also overall connectivity density *S* was found to be reduced in patients, but this effect did not reach the set statistical threshold (p = 0.062). Examination of the node-specific organizational measures, revealed a significant lower efficiency *Eff_i_* of the left precentral (q<0.05, q = FDR corrected p-value), left caudal middle frontal gyrus (q<0.05), right paracentral (q<0.05), right precuneus and posterior cingulate cortex (q<0.05). The left precentral gyrus (p = 0.042, df = 52) and right paracentral lobule (p = 0.0150) also showed a reduced level of connectivity strength *S_i_*, but these effects did not survive FDR correction.

ALSFRS or progression rate scores were not significantly correlated with network topology measures.

## Discussion

**Figure 4:**
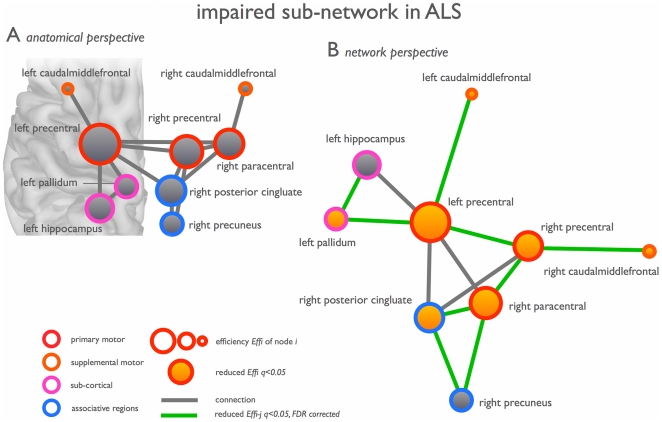
Network of impaired structural connectivity in ALS. (a) The nodes and interconnections of the NBS network (NBSa network, Figure 2a) as viewed from an anatomical perspective. (b) The NBS network from a network perspective (optimizing free Kamada-Kawai energy, constructed with pajek http://vlado.fmf.uni-lj.si/pub/networks/pajek/). Nodes and connections showing significantly reduced efficiency in patients are highlighted.

The main finding of this study is a reduced efficiency of a widespread motor connectivity network in ALS. Patients revealed a significantly impaired structural network overlapping bilateral primary motor regions (precentral gyrus and paracentral lobule, Brodmann area (BA) 4), bilateral supplementary motor regions (caudal middle frontal gyrus, BA 6), parts of the left basal ganglia (pallidum) and right posterior cingulate and precuneus (Figure 2). Specifically examining the structural topology of the NBS network using graph analysis, revealed a significant decrease in efficiency *E* of this community of regions, most pronounced in left and right primary and supplemental motor regions (Figure 4). These results suggest that ALS not only affects primary motor connections, but also the capacity of primary motor regions to connect and communicate to supplemental motor regions. In addition, motor connections to the precuneus and posterior cingulate regions, strong connected regions or hubs of the brain network [25], [42], were found to be affected in ALS. Hub regions are known to play a central role in the communication between remote brain regions [36], [43]. Taken together, our findings suggest that not only the primary motor network is affected, which tends to be the current opinion on ALS [4], [5], but that ALS may have a much more global effect on connectivity and communication efficiency of the human connectome.

As shown in Figure 3b, the impaired sub-network in patients strongly overlapped with the motor network as we found in healthy controls. Based on the central role of the primary motor regions in the NBS network (see Figure 4b), it is tempting to speculate about the idea that the disease starts in the precentral gyrus and progresses along the structural connections of the primary motor regions towards secondary motor regions. Our current study did not, however, show direct correlations of network impairment with severity or progression rate of the disease. Alternatively, brain plasticity could have potentially attributed to the reduced motor connectivity. Longitudinal and combined structural and functional MRI studies are needed to validate our hypothesis of disease progression along functional and structural connections of the motor network from primary motor regions towards secondary motor regions. Measurements of functional connectivity were not included in this study, but previous data have suggested that functional connectedness of the motor network is correlated with a faster disease progression [4].

Our finding, of degenerative effects in extra-motor regions in ALS, is in line with previous imaging studies on ALS. A number of grey and white matter voxel-based morphometry (VBM) studies [12], [44], [45], [46] as well as DTI studies [9], [11], [47], [48], [49] have reported extra-motor degenerative effects in ALS. These studies mostly resulted in an enumeration of found affected brain regions. Interpretation of this type of findings, however, is difficult and often limited to linking the function of the found regions to the clinical features in ALS. In contrast, the graph analytical network approach applied in this study provides the opportunity to examine ALS as a network disease, focusing on how the disease affects the white matter connectivity structure between brain regions. Our study may now provide a new insight into ALS, showing that the affected extra-motor regions are highly connected to the precentral gyri (primary motor regions) as the center of the degenerative process. The notion of a more widespread effect on brain connectivity, is supported by recent findings of impaired functional connectivity in patients [50], [51]. A study on resting-state fMRI data of patients with ALS showed a reduced level of functional communication in the so-called ‘default-mode network’, a clustered network overlapping medial inferior and lateral superior frontal, precuneus and inferior parietal and superior temporal brain regions [51]. This reduced default mode connectivity is in accordance with our findings of impaired structural connectedness of the motor network to the precuneus and posterior cingulate regions, key regions of this ‘default-mode network’ [14], [43].

Recent studies have reported an association between structural and functional connections in the brain network, confirming that anatomical connectivity - to some extent - bounds and shapes functional communication and connectivity between brain regions [14], [36], [42], [52], [53]. Most importantly, a recent study showed that apparent local structural effects, for example focal damage to a primary motor node, can have widespread effects on whole brain functional network communication [54]. As such, our findings of impaired structural efficiency in ALS may suggest strong functional implications for efficient information processing and integration in the motor network. Indeed, reduced levels of global efficiency and affected functional connectivity of the brain network have been reported in neurological and psychiatric brain disorders, like Alzheimer’s disease [55], [56], Parkinson [57], [58] and schizophrenia [25], [59], diseases known to affect cognitive processing. Higher levels of global brain network efficiency (and especially of frontal and temporal brain regions) are known to play a key role in cognitive functioning [19], [20], [60]. Our current results, together with recent studies examining whole-brain functional connectivity in ALS, show that ALS may affect more than simply the regions and topology of the motor network and that this underlying reduction of brain efficiency may be related to (subtle) cognitive dysfunction, observed in quite a large number of patients [61], [62], [63]. This calls for future studies including ALS patients with FTD and quantitative neuropsychological testing. Examining this sub-population in a similar graph analytical approach may provide further information on whether brain network efficiency metrics are related to an increased probability of FTD development in ALS.

A potential weakness of our study is the disproportionate number of control subjects compared to patients, as well as the lack of longitudinal measures. The study was, however, set up to examine potential effects of disability and disease progression on motor network connectivity, and therefore to include a large group of patients. The total group included less disabled patients (ALSFRS-R>40), as well as patients with major disabilities (ALSFRS-R<40). Much to our disappointment, no effects were found to correlate with either the functional impairments, disease duration or disease progression rate. In addition, turning to the methodological aspects of this study, although DTI is an established method for investigating white matter integrity, the biological substrate of FA changes is still not completely understood. Fractional anisotropy is widely used as a measure of white matter integrity, it is influenced by multiple factors, complicating a direct interpretation of the results. Well-known influences are crossing fibers, fiber re-organisation, increased membrane permeability, destruction of intracellular compartments and glial alterations [64], [65].

Second, in this study, connectivity between brain regions was taken as the average FA along a tract [25], rather than the number of streamlines interconnecting a pair of regions [42], [66]. The latter measure has been argued to be more sensitive when the impairment of a tract is relatively local, a streamline is in that case likely to terminate at the point of impairment, resulting in a reduced streamline count. This in contrast to using FA as a measure of connectivity, when local impairment may then be attenuated as the remaining part of the fiber streamline is not affected. However, this argument only holds when the impairment is severe, bringing the FA value along a point of the fiber below the stopping threshold of the tracking procedure (in this study 0.1). Based on previous DTI studies in ALS, such a severe impairment in microstructural integrity is unlikely to occur. FA alterations along the corticospinal and corpus callosal tracts - being the most extensively affected white matter tracts in ALS – appear to be rather modest (approximately 10%), despite profound motor disability among studied patients [4], [5], [47], [67]. As a result this will likely not have a strong effect on the tracing procedure; the FA value along the tracts in patients will still exceed the stopping criteria of ∼0.1, resulting in normal reconstructed tracts. Indeed, no differences were found in overall number of streamlines - overall and in corticospinal and corpus callosal tracts - in this study and in previous studies of our group [4]. Furthermore, as expected, performing a post-hoc analysis in which the NBS analysis was performed on the number of streamlines instead of FA (i.e. connectivity matrices were constructed from the (scaled) number of streamlines that could be found between region *i* and *j* in the network) did not reveal a subnetwork of affected regions related to ALS. These post-hoc results support our overall conclusion, suggesting that most fiber tracts are still present in patients with ALS, but that their microstructural organization is altered due to disease.

Third, in this study, streamline fiber tracking was used to reconstruct the connections of the brain network. The used deterministic fiber tracking algorithm (FACT) is based on having sufficient directional information at each point along the tract. When however the directional information at some point along a fiber stream is not univocal - for example due to ‘crossing fibers’ - ambiguous information on the diffusion direction may lead to low FA values, which could prematurely terminate a fiber streamline. As a result, some fiber pathways may be only partly reconstructed, leading to a reduced streamline count or some key fiber pathways may be missed at all. Alternative methods have been suggested, including tracing methods based on shortest paths [68] and probabilistic fiber tracking [69]. In addition to false negatives, due to noise in the DTI data, streamline tractography may sometimes lead to false positives, meaning the construction of incorrect streamlines, which in turn may result in an incorrect connection between region *i* and *j* in the connectivity matrix *M*. To control for the effect of false positives in the analysis, a group threshold of 2/3 was applied, stating that only connections that were present in more than 66% of both the group of patients and the group of controls were considered for analysis (see for a full description the Methods section). A useful alternative method to control for false positives, might be to include an initial streamline-threshold at the individual level, stating that only a connection between region *i* and *j* is said to be present when it consists out of more than a certain number of streamlines. Performing such an additional analysis, including a streamline threshold of 10 (i.e. taking only connections into consideration that included > = 10 streamlines, identical NBS procedure, 5000 permutations) revealed a similar affected sub-network (p<0.0030, NBS), covering the same regions as presented in Figure 2a. These additional analyses suggest that the effect of false positives to our presented results (Figure 2 and 3) is minimal. Fourth, brain networks were examined on a macroscopic scale at a relative low spatial resolution, representing the brain as a graph of 82 segmented brain regions (i.e. nodes). Recent studies have, however, suggested the use a more high-resolution approach (going up to more than a 1000 smaller regions) [39], [41], [70]. ‘Graph resolution’ has been reported to have an effect on the topological properties of brain networks [41], [71], [72].

Based on our present findings, we conclude that besides primary motor regions - as the center of the degenerative process in ALS - the motor connectome as a whole is affected in ALS including secondary motor connections. The widespread effects on the brain network were found to result from an interconnected degenerative process, suggesting that focal damage in primary motor regions in ALS may ultimately manifest in connectivity disturbances elsewhere in the brain.

## Supporting Information

Figure S1 Average connectivity distribution of the group of patients with ALS and the group of healthy controls.
(TIFF)

## References

[1] Logroscino G, Traynor BJ, Hardiman O, Chio A, Couratier P (2008). Descriptive epidemiology of amyotrophic lateral sclerosis: new evidence and unsolved issues.. J Neurol Neurosurg Psychiatry.

[2] Ngai S, Tang YM, Du L, Stuckey S (2007). Hyperintensity of the precentral gyral subcortical white matter and hypointensity of the precentral gyrus on fluid-attenuated inversion recovery: variation with age and implications for the diagnosis of amyotrophic lateral sclerosis.. AJNR Am J Neuroradiol.

[3] Turner MR, Kiernan MC, Leigh PN, Talbot K (2009). Biomarkers in amyotrophic lateral sclerosis.. Lancet Neurol.

[4] Verstraete E, van den Heuvel MP, Veldink JH, Blanken N, Mandl RC (2010). Motor network degeneration in amyotrophic lateral sclerosis: a structural and functional connectivity study.. PLoS One.

[5] Filippini N, Douaud G, Mackay CE, Knight S, Talbot K (2010). Corpus callosum involvement is a consistent feature of amyotrophic lateral sclerosis.. Neurology.

[6] Roccatagliata L, Bonzano L, Mancardi G, Canepa C, Caponnetto C (2009). Detection of motor cortex thinning and corticospinal tract involvement by quantitative MRI in amyotrophic lateral sclerosis.. Amyotroph Lateral Scler.

[7] Iwata NK, Aoki S, Okabe S, Arai N, Terao Y (2008). Evaluation of corticospinal tracts in ALS with diffusion tensor MRI and brainstem stimulation.. Neurology.

[8] Nelles M, Block W, Traber F, Wullner U, Schild HH (2008). Combined 3T Diffusion Tensor Tractography and 1H-MR Spectroscopy in Motor Neuron Disease.. AJNR Am J Neuroradiol.

[9] Sage CA, Peeters RR, Gorner A, Robberecht W, Sunaert S (2007). Quantitative diffusion tensor imaging in amyotrophic lateral sclerosis.. Neuroimage.

[10] Wong JC, Concha L, Beaulieu C, Johnston W, Allen PS (2007). Spatial profiling of the corticospinal tract in amyotrophic lateral sclerosis using diffusion tensor imaging.. J Neuroimaging.

[11] Sage CA, Van Hecke W, Peeters R, Sijbers J, Robberecht W (2009). Quantitative diffusion tensor imaging in amyotrophic lateral sclerosis: revisited.. Hum Brain Mapp.

[12] Senda J, Kato S, Kaga T, Ito M, Atsuta N (2011). Progressive and widespread brain damage in ALS: MRI voxel-based morphometry and diffusion tensor imaging study.. Amyotroph Lateral Scler.

[13] Bullmore E, Sporns O (2009). Complex brain networks: graph theoretical analysis of structural and functional systems.. Nature Reviews.

[14] van den Heuvel MP, Mandl RC, Kahn RS, Hulshoff Pol HE (2009). Functionally linked resting-state networks reflect the underlying structural connectivity architecture of the human brain.. Hum Brain Mapp.

[15] Sporns O, Zwi JD (2004). The small world of the cerebral cortex.. Neuroinformatics.

[16] Reijneveld JC, Ponten SC, Berendse HW, Stam CJ (2007). The application of graph theoretical analysis to complex networks in the brain.. Clin Neurophysiol.

[17] Kaiser M (2011). A tutorial in connectome analysis: Topological and spatial features of brain networks.. Neuroimage Epub 2011 May.

[18] Basser PJ, Pajevic S, Pierpaoli C, Duda J, Aldroubi A (2000). In vivo fiber tractography using DT-MRI data.. Magn Reson Med.

[19] van den Heuvel MP, Stam CJ, Kahn RS, Hulshoff Pol HE (2009). Efficiency of functional brain networks and intellectual performance.. J Neurosci.

[20] Li Y, Liu Y, Li J, Qin W, Li K (2009). Brain anatomical network and intelligence.. PLoS Computational Biology.

[21] Bassett DS, Bullmore ET, Meyer-Lindenberg A, Apud JA, Weinberger DR (2009). Cognitive fitness of cost-efficient brain functional networks.. Proc Natl Acad Sci U S A.

[22] Neary D, Snowden JS, Gustafson L, Passant U, Stuss D (1998). Frontotemporal lobar degeneration: a consensus on clinical diagnostic criteria.. Neurology.

[23] Cedarbaum JM, Stambler N, Malta E, Fuller C, Hilt D (1999). The ALSFRS-R: a revised ALS functional rating scale that incorporates assessments of respiratory function. BDNF ALS Study Group (Phase III).. J Neurol Sci.

[24] Jones DK, Horsfield MA, Simmons A (1999). Optimal strategies for measuring diffusion in anisotropic systems by magnetic resonance imaging.. Magn Reson Med.

[25] van den Heuvel MP, Mandl RC, Stam CJ, Kahn RS, Hulshoff Pol HE (2010). Aberrant frontal and temporal complex network structure in schizophrenia: a graph theoretical analysis.. J Neurosci.

[26] Andersson JL, Skare S, Ashburner J (2003). How to correct susceptibility distortions in spin-echo echo-planar images: application to diffusion tensor imaging.. NeuroImage.

[27] Andersson JL, Skare S (2002). A model-based method for retrospective correction of geometric distortions in diffusion-weighted EPI.. NeuroImage.

[28] Chang LC, Jones DK, Pierpaoli C (2005). RESTORE: robust estimation of tensors by outlier rejection.. Magn Reson Med.

[29] Beaulieu C, Allen PS (1994). Determinants of anisotropic water diffusion in nerves.. Magn Reson Med.

[30] Basser PJ, Pierpaoli C (1996). Microstructural and physiological features of tissues elucidated by quantitative-diffusion-tensor MRI.. Journal of Magnetic Resonance.

[31] Mori S, Crain BJ, Chacko VP, van Zijl PC (1999). Three-dimensional tracking of axonal projections in the brain by magnetic resonance imaging.. Annals of Neurology.

[32] Mori S, van Zijl PC (2002). Fiber tracking: principles and strategies - a technical review.. NMR Biomed.

[33] Mori S, Kaufmann WE, Davatzikos C, Stieltjes B, Amodei L (2002). Imaging cortical association tracts in the human brain using diffusion-tensor-based axonal tracking.. Magn Reson Med.

[34] Mandl RC, Schnack HG, Zwiers MP, van der Schaaf A, Kahn RS (2008). Functional diffusion tensor imaging: measuring task-related fractional anisotropy changes in the human brain along white matter tracts.. PLoS ONE.

[35] Mandl RC, Schnack HG, Luigjes J, van den Heuvel MP, Cahn W (2010). Tract-based Analysis of Magnetization Transfer Ratio and Diffusion Tensor Imaging of the Frontal and Frontotemporal Connections in Schizophrenia.. Schizophr Bull.

[36] Van den Heuvel MP, Mandl RC, Luigjes J, Hulshoff Pol HE (2008). Microstructural organization of the cingulum tract and the level of default mode functional connectivity.. J Neurosci.

[37] Fischl B, Sereno MI, Dale AM (1999). Cortical surface-based analysis. II: Inflation, flattening, and a surface-based coordinate system.. Neuroimage.

[38] Fischl B, van der Kouwe A, Destrieux C, Halgren E, Segonne F (2004). Automatically parcellating the human cerebral cortex.. Cereb Cortex.

[39] Hagmann P, Kurant M, Gigandet X, Thiran P, Wedeen VJ (2007). Mapping human whole-brain structural networks with diffusion MRI.. PLoS ONE.

[40] Zalesky A, Fornito A, Bullmore ET (2010). Network-based statistic: identifying differences in brain networks.. Neuroimage.

[41] Rubinov M, Sporns O (2010). Complex network measures of brain connectivity: uses and interpretations.. Neuroimage.

[42] Hagmann P, Cammoun L, Gigandet X, Meuli R, Honey CJ (2008). Mapping the Structural Core of Human Cerebral Cortex.. PLoS Biology.

[43] Greicius MD, Supekar K, Menon V, Dougherty RF (2008). Resting-State Functional Connectivity Reflects Structural Connectivity in the Default Mode Network.. Cereb Cortex.

[44] Thivard L, Pradat PF, Lehericy S, Lacomblez L, Dormont D (2007). Diffusion tensor imaging and voxel based morphometry study in amyotrophic lateral sclerosis: relationships with motor disability.. J Neurol Neurosurg Psychiatry.

[45] Agosta F, Pagani E, Rocca MA, Caputo D, Perini M (2007). Voxel-based morphometry study of brain volumetry and diffusivity in amyotrophic lateral sclerosis patients with mild disability.. Hum Brain Mapp.

[46] Kassubek J, Unrath A, Huppertz HJ, Lule D, Ethofer T (2005). Global brain atrophy and corticospinal tract alterations in ALS, as investigated by voxel-based morphometry of 3-D MRI.. Amyotroph Lateral Scler.

[47] van der Graaff MM, Sage CA, Caan MW, Akkerman EM, Lavini C (2011). Upper and extra-motoneuron involvement in early motoneuron disease: a diffusion tensor imaging study.. Brain.

[48] Agosta F, Pagani E, Petrolini M, Caputo D, Perini M (2010). Assessment of white matter tract damage in patients with amyotrophic lateral sclerosis: a diffusion tensor MR imaging tractography study.. AJNR Am J Neuroradiol.

[49] Senda J, Ito M, Watanabe H, Atsuta N, Kawai Y (2009). Correlation between pyramidal tract degeneration and widespread white matter involvement in amyotrophic lateral sclerosis: A study with tractography and diffusion-tensor imaging.. Amyotroph Lateral Scler.

[50] Jelsone-Swain LM, Fling BW, Seidler RD, Hovatter R, Gruis K (2010). Reduced Interhemispheric Functional Connectivity in the Motor Cortex during Rest in Limb-Onset Amyotrophic Lateral Sclerosis.. Front Syst Neurosci.

[51] Mohammadi B, Kollewe K, Samii A, Krampfl K, Dengler R (2009). Changes of resting state brain networks in amyotrophic lateral sclerosis.. Exp Neurol.

[52] Honey CJ, Kotter R, Breakspear M, Sporns O (2007). Network structure of cerebral cortex shapes functional connectivity on multiple time scales.. Proc Natl Acad Sci U S A.

[53] Honey CJ, Sporns O, Cammoun L, Gigandet X, Thiran JP (2009). Predicting human resting-state functional connectivity from structural connectivity.. Proc Natl Acad Sci U S A.

[54] Alstott J, Breakspear M, Hagmann P, Cammoun L, Sporns O (2009). Modeling the impact of lesions in the human brain.. PLoS Comput Biol.

[55] Stam CJ, de Haan W, Daffertshofer A, Jones BF, Manshanden I (2009). Graph theoretical analysis of magnetoencephalographic functional connectivity in Alzheimer's disease.. Brain.

[56] Chen G, Ward BD, Xie C, Li W, Wu Z (2011). Classification of Alzheimer Disease, Mild Cognitive Impairment, and Normal Cognitive Status with Large-Scale Network Analysis Based on Resting-State Functional MR Imaging.. Radiology.

[57] Wu T, Wang L, Chen Y, Zhao C, Li K (2009). Changes of functional connectivity of the motor network in the resting state in Parkinson's disease.. Neurosci Lett.

[58] van Eimeren T, Monchi O, Ballanger B, Strafella AP (2009). Dysfunction of the default mode network in Parkinson disease: a functional magnetic resonance imaging study.. Arch Neurol.

[59] Liu Y, Liang M, Zhou Y, He Y, Hao Y (2008). Disrupted small-world networks in schizophrenia.. Brain.

[60] Achard S, Bullmore E (2007). Efficiency and cost of economical brain functional networks.. PLoS Computational Biology.

[61] Ringholz GM, Appel SH, Bradshaw M, Cooke NA, Mosnik DM (2005). Prevalence and patterns of cognitive impairment in sporadic ALS.. Neurology.

[62] Raaphorst J, de Visser M, Linssen WH, de Haan RJ, Schmand B (2010). The cognitive profile of amyotrophic lateral sclerosis: A meta-analysis.. Amyotroph Lateral Scler.

[63] Gordon PH, Goetz RR, Rabkin JG, Dalton K, McElhiney M (2010). A prospective cohort study of neuropsychological test performance in ALS.. Amyotroph Lateral Scler.

[64] Acosta-Cabronero J, Williams GB, Pengas G, Nestor PJ (2010). Absolute diffusivities define the landscape of white matter degeneration in Alzheimer's disease.. Brain.

[65] Beaulieu C (2002). The basis of anisotropic water diffusion in the nervous system - a technical review.. NMR in Biomedicine.

[66] Zalesky A, Fornito A, Seal ML, Cocchi L, Westin CF (2011). Disrupted axonal fiber connectivity in schizophrenia.. Biol Psychiatry.

[67] Agosta F, Rocca MA, Valsasina P, Sala S, Caputo D (2009). A longitudinal diffusion tensor MRI study of the cervical cord and brain in amyotrophic lateral sclerosis patients.. J Neurol Neurosurg Psychiatry.

[68] Zalesky A (2008). DT-MRI fiber tracking: a shortest paths approach.. IEEE Trans Med Imaging.

[69] Behrens TE, Woolrich MW, Jenkinson M, Johansen-Berg H, Nunes RG (2003). Characterization and propagation of uncertainty in diffusion-weighted MR imaging.. Magn Reson Med.

[70] van den Heuvel MP, Stam CJ, Boersma M, Hulshoff Pol HE (2008). Small-world and scale-free organization of voxel-based resting-state functional connectivity in the human brain.. Neuroimage.

[71] Fornito A, Zalesky A, Bullmore ET (2010). Network scaling effects in graph analytic studies of human resting-state FMRI data.. Front Syst Neurosci.

[72] van den Heuvel MP, Hulshoff Pol HE (2010). Exploring the brain network: a review on resting-state fMRI functional connectivity.. Eur Neuropsychopharmacol.

