# Supplementary figures and images for: Impaired Structural Motor Connectome in Amyotrophic Lateral Sclerosis
Source: PLoS One. 2011 Sep 2;6(9):e24239. doi: 10.1371/journal.pone.0024239 (PMC3166305; doi:10.1371/journal.pone.0024239)

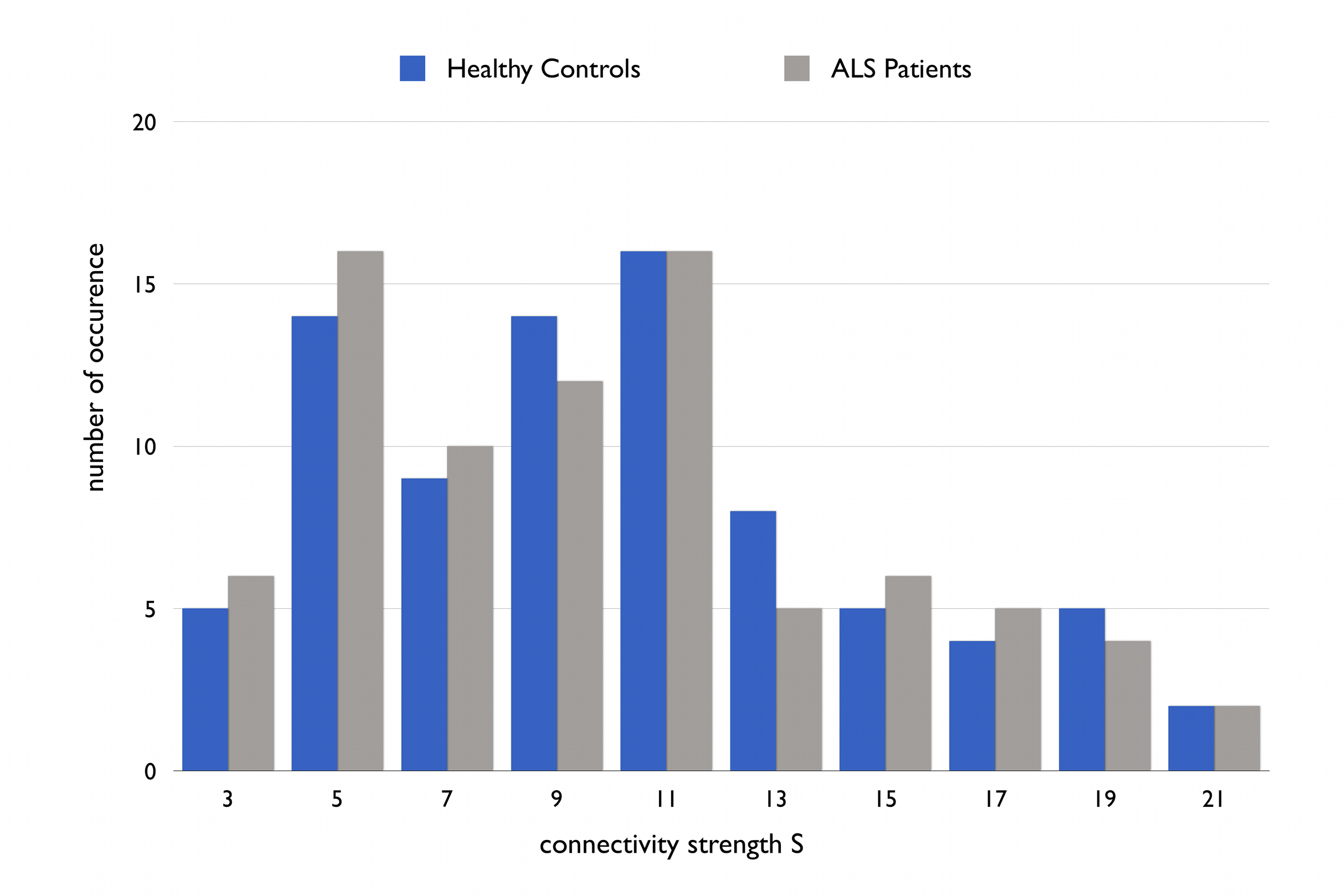

Supplement: Figure S1 — Average connectivity distribution of the group of patients with ALS and the group of healthy controls. (TIFF) [file pone.0024239.s001.tiff]
